# Supplementary material for: Gene expression profiling of the masticatory muscle tendons and Achilles tendons under tensile strain in the Japanese macaque Macaca fuscata
Source: PLoS One. 2023 Jan 19;18(1):e0280649. doi: 10.1371/journal.pone.0280649 (PMC9851512; doi:10.1371/journal.pone.0280649)
Supplement: Attachment — Submitted filename: revision2_Response_to_reviewers.docx [file pone.0280649.s007.docx]

Response to reviewers

We thank the Editor and the Reviewers for their insightful and helpful comments. We have tried to address all issues raised by the reviewers. Also, we have thoroughly revised the manuscript. We highlighted the corrected or additional words/sentences with red font and the proofread words/sentences with the underline.

*Review Comments to the Author*

*Reviewer #2: The authors are to be commended on an interesting paper that demonstrates differences between functionally distinct load-bearing tendons. The authors have made significant improvements in this review however I found the discussion somewhat superficial and the meaning and relevance of the findings (aside from the fact that they are observationally different) in a wider context is not explored. What might the differences mean in terms of clinical relevance or function?*

Author’s Response: Thank you very much for the valuable comments. We have added the following sentences in the Discussion section (line 315-332): “Normally, the force applied to the masticatory tendon during chewing is approximately 320 N, and the force applied to the Achilles tendon during walking is approximately 1,000 N [38][39]. It is assumed that the Achilles tendon has high load-bearing properties. On the other hand, it has been reported that patients with MMTAH show hyperplasia of the masticatory muscle tendon without hyperplasia of the Achilles tendon, but the masticatory muscle tendon is subjected to a bite force of approximately 1,000 N [40]. Given these facts, it is consistent to assume that different genes are expressed in masticatory muscle tendons, which are considered vulnerable to high loads, than in Achilles tendons, which can withstand high loads. Although high loads were applied to each tendon cell in this study, different genes were expressed in the masticatory muscle tendon and Achilles tendon, respectively. In masticatory muscle tendon cells, which are vulnerable to high loads, the expression of genes that promote cell cycle progression and cytoskeletal reorganization (TUBB3 and SEPTIN9) and genes important for tendon regenerative capacity (SPHK1 and GPNMB) were elevated. We speculate that the faster turnover in highly loaded masticatory muscle tendon cells may increase the speed of cell regeneration, resulting in hyperplasia of tendon tissue. In other words, differences in gene expression between masticatory muscle tendons and Achilles tendons may be associated with differences in vulnerability to loading.”

We have added the following references.

[38] Lundgren D, Laurell L. Occlusal force pattern during chewing and biting in dentitions restored with fixed bridges of cross-arch extension. I. Bilateral end abutments. J Oral Rehabil. 1986 Jan;13(1):57-71.

[39] Fröberg A, Komi P, Ishikawa M, Movin T, Arndt A. Force in the achilles tendon during walking with ankle foot orthosis. Am J Sports Med. 2009 Jun;37(6):1200-7.

[40] Fujii T, Watanabe M, Motohashi T, Kubo H, Ohnishi Y, Shirao K, Nakajima M. Efficacy of surgical treatment for masticatory muscle tendon­aponeurosis hyperplasia. J Osaka Dent Univ 2020; 54 (2) : 213−218
